# Perceptions of Patient Engagement Applications During Pregnancy: A Qualitative Assessment of the Patient’s Perspective

**DOI:** 10.2196/mhealth.7040

**Published:** 2017-05-26

**Authors:** Maren Goetz, Mitho Müller, Lina Maria Matthies, Jenny Hansen, Anne Doster, Akos Szabo, Jan Pauluschke-Fröhlich, Harald Abele, Christof Sohn, Markus Wallwiener, Stephanie Wallwiener

**Affiliations:** ^1^ Department of Obstetrics and Gynecology University of Heidelberg Heidelberg Germany; ^2^ Department of Psychology Ludwig Maximilian University Munich Germany; ^3^ Department of Obstetrics and Gynecology University of Tübingen Tübingen Germany

**Keywords:** pregnancy, telemedicine, mobile applications, information procurement, patient participation, qualitative research

## Abstract

**Background:**

With growing demand for medical information and health applications in pregnancy, the potential of electronic health (eHealth) and mobile health (mHealth) solutions in clinical care is increasingly unfolding. However, we still do not know how pregnant women engage with mobile apps, how such apps impact routine medical care, and whether benefit expectations are met. Whereas recent research has raised the subject of user distribution and analyzed the content of pregnancy applications, there is still a significant knowledge gap regarding what pregnant women like and dislike about pregnancy tools, along with how such interventions could be improved.

**Objective:**

The aim of the study was to examine the perceptions and expectations of mobile and Web-based patient-engagement pregnancy applications. We assessed usability requirements, general acceptance of eHealth, and the impact of eHealth and mHealth pregnancy applications on the doctor-patient interaction and daily clinical routine.

**Methods:**

A qualitative study was conducted at the maternity department of a major German university hospital. The sample included 30 women with low- to medium-risk pregnancies. Half of the patients were seen during outpatient care and half were hospitalized for several days. The extent and frequency of Web- and mobile phone app usage were assessed. Semistructured interviews were conducted and analyzed using systematic thematic analysis.

**Results:**

Patients had a high demand for Web-based pregnancy applications. Study findings suggested a strong request for personalization, monitoring, and accessibility for frequent use as main themes derived from the interviews. Fostering patient empowerment in the doctor-patient relationship was also highly valued for a pregnancy app. Participants favored further integration of medical apps in their daily routine and pregnancy care. However, concerns were raised about content quality, trustworthiness of Web sources, and individual data security.

**Conclusions:**

eHealth and mHealth applications are a highly frequented source of information. Expectations and usability requirements for those applications are also high, thus posing a challenge to interdisciplinary service providers. Patients’ attitude toward integrating apps in routine care settings was positive with a favorable influence on patient empowerment. Health care professionals should guide pregnant women toward a successful integration of these educational tools in pregnancy care.

## Introduction

With patients’ growing demand for medical information and, at the same time, the rapidly evolving opportunities for health care providers to integrate and adopt information technology, the potential of electronic health (eHealth) and mobile health (mHealth) solutions is increasingly unfolding [1]. Here, eHealth is seen as the interface of medical informatics, health care research, and health services, offered preferably via the Web or mobile technology [2,3]. mHealth encompasses the use of mobile communication and multimedia, and their integration in wireless health care delivery systems [4,5]. There is growing evidence that such applications and interventions enhance the doctor-patient connection toward a more partner-like relationship, leading to “patient empowerment” and “patient engagement” [6-11]. Providing evidence-based information and Web-based access to electronic health reports could open new pathways for informing patients. In addition to the omnipresence and penetration of mobile phones in our society, these options have the potential to avail mHealth for prenatal and newborn care, especially in developing economies [12].

Women of childbearing age frequently use the Web and mobile phone apps as a source of information [13,14]. In addition, pregnant women, particularly in developed nations, are using social media to search for information about pregnancy, birth, and breastfeeding [15]. Women conduct Web-based research regularly, most often in the early stages of pregnancy when they have recently entered into a new and possibly frightening life situation [16]. Over and above other major benefits such as anonymity, simplicity, and rapidity, the major reason for searching the Web is the explicit need for advanced knowledge on a wide range of pregnancy-related topics [17]. Moreover, pregnant women are sharing their experiences and knowledge through online communities with other mothers-to-be [17,18]. Interaction in online discussion forums influences maternal health literacy through increased awareness of health promotion and health-related knowledge. For some, the information provided by other pregnant women is valued more highly than advice from health care professionals [19], thus underlining the need for high-quality and evidence-based health information on the Web.

Recent studies on the use of eHealth during pregnancy showed that most participants trusted Web-based information. However, the major proportion of these users had none to little knowledge of websites run by nonprofit organizations [13,16]. Bernhardt et al showed that mothers of young children mainly accessed commercial websites for health information, but at the same time expressed disdain for commercial websites [20].

A closer look at the increasing use of mobile pregnancy apps presents an ambiguous picture: app developers combine claims of evidence-based expertise with attempts to engage patients as part of their promotional efforts [21,22]. Yet, many users do not actively assess content validity or consider privacy issues regarding personal data collected by these apps. It is undeniable that a significant proportion of medical websites and the majority of mHealth apps are not transparent regarding information sources and privacy policy [23]. It is important, therefore, that health care professionals and pregnant women are aware of these influencing factors, their benefits, and their limitations [24]. Major providers such as Apple recently took steps to tighten requirements for medical apps (ie, more stringent data protection regulations) [25].

Notwithstanding the potentially negative connotations, the use of Web and mHealth apps undoubtedly opens new spaces for future progress. Several studies focusing on the use of eHealth and mHealth during the prenatal period demonstrated increased patient satisfaction and engagement regarding weight and blood pressure control during pregnancy [11,26-28], breastfeeding over time [29], and enhancement of subjective well-being and self-management [10,30] through the use of digital monitoring systems.

Whereas the use, the user characteristics, and the content of pregnancy-related apps are well described [14,17,31,32], qualitative studies focusing on the patients’ perspective are sparse. Knight-Agarwar et al described the process of developing and pilot testing a mobile app to monitor gestational weight gain. Participants found the app motivational and approved of the nutritional information, but criticized usability [33]. Two very recent Australian studies investigated the use and value of digital media and pregnancy apps, including attitudes toward the information provided, required features (apps as tracking device or photo storage), and reservations on data protection [22,34]. The authors found that pregnant women placed high value on the information and support received through Web-based sources and apps; yet, considerations on content validity or issues concerning data security varied.

Despite the increasing influence of the Web and the rapid evolution of mHealth technology, little has changed in the prenatal care visit structure over the past years. In addition, we still do not know how pregnant women engage with mobile technology, how these mobile tools affect medical care, and whether the apparent benefits they promise are provided [21,35,36].

To fill this gap, this trial aimed to analyze (1) perceptions and requirements for pregnancy apps from the patients’ perspective and (2) their impact on daily clinical routine by using qualitative research methods. We specifically decided to use a qualitative approach to obtain an unfiltered, unbiased impression of what really matters to pregnant women. In particular, we focused on the question of what features such apps must provide to meet the patients’ expectations, and thus improve pregnancy care.

## Methods

### Sample

**Figure 1:**
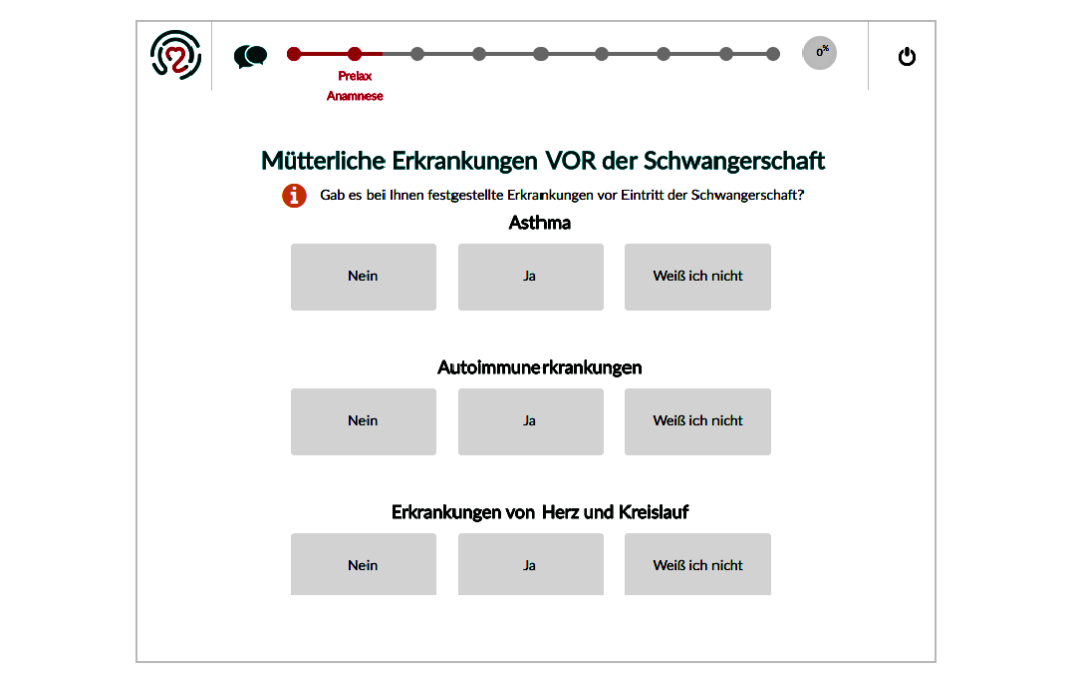
Electronic data capture (eDC) tool for patient reported outcome (PRO) data.

**Figure 2:**
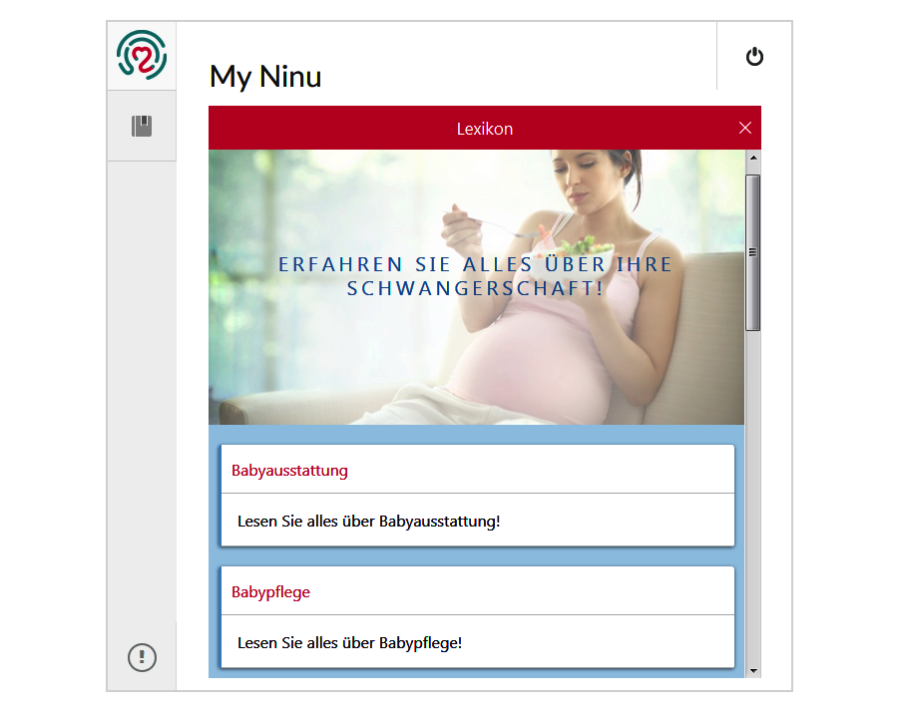
Patient education application “lexicon”.

A mixed-method study with quantitative and qualitative approaches was carried out among pregnant women who attended prenatal care at the university hospital of Heidelberg from May to July 2016, a perinatal center of the highest level providing health services to low, medium, and high-risk obstetrical patients, and performing over 2000 deliveries per year. Criteria for eligibility included age of 18 years or older and a sufficient knowledge of the German language. In total, 37 randomly selected pregnant women were asked to participate, 30 of whom agreed. Reasons for not participating included lack of time or interest or poor physical condition. The sample consisted of healthy and low- to medium-risk patients to detect different user preferences and needs. Half of the group were seen during outpatient care (n=15) and half were hospitalized for the risk of preterm birth (n=15). No acute or high-risk patients were included in the study. No compensation was offered to the participants. The participants completed self-administered questionnaires on medical data, sociodemographics, and private use of technologies, developed and validated by an expert panel of doctors and midwives. Semistructured interviews were carried out with each patient to gain detailed insight in user perceptions of eHealth solutions in general after demonstrating the purpose of the questions based on a very basic patient engagement pregnancy application (PRELAX). This Web-based application consisted of a simple survey tool comprising several digital questionnaires and an information tool covering important evidence-based, pregnancy-related topics, such as nutrition, sports, body care, and nursing (Figures 1 and 2). Ethics approval was granted by the ethical committee of the University of Heidelberg.

### Measurements

We gathered medical and sociodemographic data such as marital status, education, and level of employment. Medical data included current diagnoses, previous pregnancies, miscarriages, and number of live-births and were double-checked against the hospital records. The private use of technologies was assessed based on individual use duration and frequency. Prior usage of the Web for information acquisition and interest in pregnancy eHealth applications or mobile apps were also assessed by the question: “Have you ever searched the Internet or a mobile app for specific questions concerning your pregnancy/hospital/clinical examinations?” Additionally, the participants were asked to complete a questionnaire focusing on the technical and graphical implementation and user-friendliness of pregnancy applications in general. The acceptance of introducing eHealth applications in clinical health care and the conceivable impact on the patients’ health status, treatment, and quality of life was also assessed.

Apart from quantitative measurements, we used qualitative methods to gain a deeper understanding of the patients’ view through first-hand experience and truthful quotations of semistructured face-to-face interviews. Those interviews were carried out by a trained interviewer under the supervision of a senior physician with expertise in the field of perinatology and prenatal diagnostics. The participants were aware that the interviewer was connected with the hospital but was at no time involved in their personal health care. Moreover, women were reassured about confidentiality and encouraged to openly criticize and provide lively feedback on the care they had received. After giving written consent, the interviews were digitally recorded and lasted 37 min on average (34-43 min). The interview guideline consisted of open-ended questions to assess (1) user satisfaction with the patient engagement pregnancy application developed and (2) usability and personal requirements for the use of eHealth and mHealth services (see Textbox 1 “interview section”).

The interviewer was free to follow spontaneous lines of thought through a flexible use of the interview schedule. Finally, the interviews were transcribed and all personal, identifying data removed.

### Data Analysis

Audio-recorded interviews were transcribed verbatim and verified against the actual recordings by the authors. The authors reviewed these audio recordings for completeness of captured data in the notes. Transcripts were analyzed using systematic qualitative thematic analysis [37]. First, this approach began with familiarization with the data. Key issues and initial codes were identified, edited, and grouped into emerging themes to form the basis of the coding framework. In relation to the original data, these codes and themes were refined to ensure theoretical connectedness [38] and were finally defined. The study team discussed each stage of the analysis process to ensure correctness of the themes and their supporting data. We used interrater reliability, constant data comparison, and proper audit trail of material and processes as validation strategies. Quotes that reflected the various findings from the original data were collected for the manuscript (abbreviations: patient [P]; ambulatory [A], stationary [S]) and translated into English. Qualitative analysis software (QDA Miner Lite 1.4.5) was used to facilitate data organization using coding frequency, retrieval, and filter functions.

**Interview section.**

Questions used to guide the interviews

- Do you think the digital survey was adequately tailored to your specific situation or would you rather have different questions?
- Considering the graphic design of the application presented: what did you like, what would you like to change?
- What should pregnancy applications provide to meet pregnant women’s expectations and to improve pregnancy care?
- What kind of incentives must be offered to pregnant women to use Web-based or mobile pregnancy applications regularly?
- What do you think about using applications surveying your medical care, health status, or quality of life (ie, mobile apps, Web-based portals) regularly in clinical routine?

## Results

### Demographics

In all, 30 pregnant women were included in the final study sample. Mean (standard deviation [SD]; range) maternal age was 33 years (3.4; 27-40) and mean gestational age was 33 weeks (4.3; 25-40). Demographic and pregnancy-related characteristics of the study population are shown in Table 1. The most common diagnoses of the participants are listed in Table 2.

Regarding information procurement, 26 women (87%, 26/30) frequently used Web sources to gather information on pregnancy-related topics whereas 18 women (60%, 18/30) additionally used a mobile pregnancy app. Furthermore, 24 women sought information on prenatal checkups (80%, 24/30) and 19 participants searched the Web for local maternity hospitals (63%, 19/30). Only 5 women used online communities regularly. In all, 87% (26/30) showed interest in patient engagement applications and were willing to complete health surveys electronically or via mobile devices in the future. Misgivings about the use of digital surveys in daily routine care included preference of personal treatment (n=4), concerns regarding data security (n=3), and others (n=1). A total of 22 women believed that introducing digital health surveys would improve clinical health care (73%, 22/30), 2 women assumed possible deterioration, and 6 women expected no effect at all (20%, 6/30).

### Interview Findings

After analyzing data, three key themes emerged: (1) demanding expectations and perceptions for Web-based pregnancy applications, (2) favorable impact on doctor-patient relationship, and (3) frequent use and challenging requirements of eHealth applications during pregnancy. To quantify these findings, the number of counts and the coding frequency are indicated in brackets whenever possible and appropriate.

**Table 1:** Sample characteristics (N=30).

Characteristics		Frequency	n (%)
**Education level**			
	Low secondary education	1	1 (3)
	High secondary education	8	8 (27)
	Advanced technical college entrance qualification	5	5 (17)
	University entrance qualification	16	16 (53)
	Total	30	30 (100)
**Social status**			
	Married and living together	22	22 (73)
	Married but living apart	1	1 (3)
	Single	6	6 (20)
	Divorced	1	1 (3)
	Total	30	30 (100)
**Level of employment**			
	Full-time	17	17 (57)
	Part-time	9	9 (30)
	Temporary exempted	4	4 (13)
	Total	30	30 (100)
**Current professional position**			
	Employee	25	25 (83)
	Civil servant	5	5 (17)
	Total	30	30 (100)
**Gravidity**			
	First pregnancy	10	10 (33)
	Second	11	11 (37)
	Third or more	9	9 (30)
	Total	30	30 (100)

**Table 2:** Diagnoses (N=30; multiple answers possible).

Diagnoses	Frequency	n (%)
Uneventful pregnancy	3	3 (10)
Cervical insufficiency	11	11 (37)
Preterm labor	6	6 (20)
Gestational diabetes	4	4 (13)
Placenta previa	3	3 (10)
Status after Caesarean section	3	3 (10)
Preeclampsia	3	3 (10)
Vaginal bleeding	2	2 (7)
Others	7	7 (23)

### Expectations and Perceptions for Web-Based Pregnancy Applications

#### Evidence-Based Information

Most pregnant women criticized the lack of scientifically validated Web sources about relevant topics such as fetal development, nutrition, or pregnancy-related complications. From the patients’ perspective, reliable information such as patient centered medical guidelines should be available on the Web to prevent uncertainty.

...because there is so much information out there, and when you get some sort of guideline, just like yours (relating to the PRELAX app.) Then that’s something different from reading magazines or other Web-based portals.

S11

Health education also played a major role for many participants (16/30; 53%), particularly for women with a low educational level or missing medical background.

(It would be important) that the patients were more informed...Because I have the feeling that many pregnant women, especially the less educated, just know too little about their pregnancies...Maybe through answering the (survey) questions the women might experience a kind of wow-effect.

A14

The demand for information was high, in both the inpatient and outpatient setting. The need for solid background information through pregnancy Web-based applications and mobile apps was of major priority.

#### Personalization

Furthermore, the participants missed a certain personal touch in most Web-based applications (11/30; 37%). A personal welcoming message and the possibility to upload pictures such as fetal ultrasound images were attractive for many women.

Or perhaps...adding a little personal touch. That you do the log-in and then you read: “Hello, nice to have you back, Mrs. Smith”. And, I don’t know..., maybe also a profile and pictures, something like that...Then it would be more individual and thus more interesting for the women...

A10

For some participants it was difficult to cope with unexpected pregnancy complications such as miscarriages or preterm labor and many would have liked to share as much information as possible.

You would like to transmit personal information, indeed; especially...issues that weigh heavily on your mind...So that you have a stronger personal link to it.

S2

Overall, many participants assumed that the willingness to use Web-based applications regularly would increase significantly, if they were more related to the users’ personal concerns.

#### The Role of Artificial Intelligence and Individualized Feedback Algorithms

Additionally, more than one-third of the pregnant women (11/30; 37%) expected individualized feedback algorithms via artificial intelligence software on the data they entered, either as a score or an interactive graphic. Furthermore, many would have liked to receive immediate replies to conspicuous answers and practical advice on how to proceed when experiencing specific conditions.

That one could simply say: “Oh, the patient’s blood pressure is relatively high” and then, I don’t know, just a few simple measures, how to lower it...This would add tremendous value for most women.

A10

#### Interaction: Community-Based Features

Most pregnant women found features to interact and exchange views with other mothers very useful.

So, what I’ve noticed among my friends: usually women want to share experiences, make comparisons...Just like: “Well, the doctor has told me recently that my baby weighs about 2.5 kg in the ultrasound measurement. Is this actually normal?

A10

Therefore, a common request was standard integration of communication platforms in pregnancy applications. In contrast to already existing online pregnancy forums, these communication tools should be open for lay people and medical professionals who could then be contacted directly via a chat feature.

Somehow there should be a chat room, where a doctor is available or other medical staff. So, one could ask...Specific questions...and you might even get a response from a professional.

A8

#### Usability Requirements

As many participants were frequent mobile phone (25/30; 83%) and tablet (14/30; 47%) users, usability requirements were consistently high. While pilot testing the new PRELAX application, most women criticized nonfunctional buttons or slow loading of a page (16/30; 53%).

The pages must load within seconds. Otherwise it’s obviously really annoying...When the pages don’t load quickly, then you might eventually get tired.

S11

In general, all women preferred an easy-to-use interface in Web-based applications and did not want to be held up with time-consuming technical issues.

### Impact on Doctor-Patient Relationship

Before exploring the influence of using eHealth solutions on the doctor-patient interaction, the participants were asked for their perceptions of the current clinical routine.

In the experience of many women (9/30; 30%), doctors did not take enough time for direct doctor-patient interaction; especially time spent on rounds or conversations was considered far too short. In addition, some women felt that their physicians did not get involved enough with their issue and the average self-perceived knowledge on the patient records was rated as insufficient.

At the beginning it was a bit tricky here: there were always a lot of different doctors who then said: “Hum, sorry, but what exactly did you have?” It was a bit exhausting for me to explain this again and again, and then: “Oh, sure, that’s what you had!”

S12

Another repeatedly mentioned concern (8/30; 27%) was that women could not discuss their questions due to time-pressure and their worries about clinical examinations.

Usually the consultation goes by really quickly. Most of the time you are so excited and nervous thinking: “How’s my little baby?” that, after you have left the hospital, you notice: “Oh, I forgot to ask this or that once again.”

S14

To solve this issue, several women (16/30; 53%) suggested using medical applications or Web portals to be prepared for the consultation. A printed medical report summarizing the most important facts could then guide the patients through the conversation with their physician.

For example, if you use medically-validated applications right before the medical consultation, then I think it’s great; because you simply have a good basis for what you want to discuss with your doctors. During the conversation you probably won’t forget that much then.

A10

Interpersonal relations and face-to-face conversations played an outstanding role in the doctor-patient relationship, especially for many inpatients (12/30; 40%). Several participants (8/30; 27%) stated that a Web-based pregnancy application, however good it may be, could never replace individual medical care.

...what I have also noticed in those apps: if you are asked...to say how you feel...That’s always just a matter of interpretation, for example what “good” really means to you. This is an initial assessment to know where you stand. But it definitely cannot replace a private conversation—for me, this is still extremely important.

A7

However, the participants approved of integrating new technologies in clinical routine as an excellent addition (7/30; 23%).

I like this principle because...I know exactly, that via tablet one would admit things you wouldn’t necessarily tell the doctor or nurse. So, for starters, you can state it in the application. Of course, a conversation shouldn’t be missed afterwards, but this might make it easier for you to overcome yourself.

S14

The majority of women (26/30; 87%) wanted applications to be implemented in routine pregnancy care in order to detect and prevent serious pregnancy conditions already at an early stage.

### Use and Requirements of eHealth Applications During Pregnancy

#### Information Procurement

For most women (26/30, 87%), the Web was considered the major source of information, providing information quickly and easily. However, some participants criticized the deficient quality of Web resources, in particular online communities for future mothers. Several women pointed out that after having read forum entries they felt insecure and confused.

If you have a certain problem, you quickly start reading these online forums...I think sometimes this is not so good, because what you read can be a bit unsettling.” Interviewer: “Is it also important for you to know who provides the information?” P: “Actually yes!...If you hear the same thing from a doctor, it’s quite different to random women writing about their stuff (laughs).”

A11

Thus, some participants used Google to look up pregnancy symptoms they were experiencing, but would not recommend it.

Several participants were already using mobile pregnancy apps regularly (18/30; 60%). They explicitly liked to retrieve suitable information about relevant pregnancy topics on a weekly basis, hence, not too much information at once. For many women, mobile phone apps were considered to be playful but also useful tools.

I think pregnant women are more likely to use a mobile app..., which assesses various issues weekly. So, you know, one could feel somehow accompanied medically. But it wouldn’t be too much information all at once. I think those technical solutions would be attractive for pregnant women.

S2

#### Attitude Toward Digitization

In general, many participants (18/30; 60%) had a positive attitude toward the growing eHealth movement and could imagine a more substantial role for applications in daily routine. For example, one woman suggested connecting mobile lifestyle apps with medical applications to gather as much data as possible.

I really think this is appropriate for our age. I mean, if you look around these days, there is an app for everything!!...And that is also the trend for the future. Especially in the medical fields such tools would be great with already existing standard lifestyle apps. It would be nice, if you combined these maybe. Then you would have additional parameters or data on the general health, sleep patterns, nutrition...

S10

Furthermore, sharing data among health care professionals (ie, physicians and midwives) or even health insurance companies through standardized networks constituted a major request (7/30; 23%).

What you mentioned earlier: that you could forward your data directly to your gynecologist, midwife, hospital etc., and they equally have access to the patient’s data, for example. In a digital age like ours that would probably be very useful. Then you wouldn’t have to fill out another form each time.

A7

Some women also hoped that the technical advantages might enable faster interventions in case of any critical pregnancy condition. Although most participants appreciated the implementation of applications in pregnancy care because of their easy-use and ubiquitous availability, there was disagreement on the handling and transfer of personal data.

#### Data Security

Notably, hospitalized women (6/30; 20%) were worried about unauthorized third-party accesses to their stored medical data.

Of course, on the other hand one is always afraid that the personal data might go anywhere, and there is also the risk of unauthorized accesses to your data you actually do not agree with.

S5

Various women (8/30; 27%) expressed concerns about data security, especially in the field of mobile apps, since many free apps make private data easily accessible.

As I said, I’m very critical about patient data in general, especially in terms of data security...If you have a free app, it really depends on what happens to the private data. As a matter of fact, usually the information is stored on the app itself, and so other apps might gain access to the data easily.

A5

Despite these misgivings, several women were willing to transmit their personal data at the touch of a button.

Of course...it would seem reasonable that a considerable amount of data could be forwarded to different recipients by using such tools. Also, my attitude towards data security isn’t like: “For God’s sake: Cannot be, must not be!” Because, I think that only those will have access to the data who should have. So, I do believe that such things might facilitate certain procedures a lot.

A12

#### Personal Incentive

For many participants (11/30; 37%), there was no need to offer special incentives to use eHealth applications. However, some women pointed out that they would not have used an application surveying their pregnancy course from home if they had not shown any symptoms.

Personally I would be satisfied, if I could obtain information about my particular situation...But, to be honest, if I were completely healthy and had an uncomplicated pregnancy from the beginning to the end, then my incentives here would be rather low. Then, I would probably need something else, maybe like individual health care procedures, that kind of thing.

A6

In conclusion, most women (20/30; 70%) wished to benefit from the new technological solutions and take best advantage of them for the course of their pregnancy. Particularly the outpatient group stated that using such applications wasn’t seen as an end in itself and they were willing to contribute to implementing pioneering eHealth applications (6/30; 20%).

## Discussion

### Principal Findings

In our study, the following main requirements of eHealth applications for pregnancy emerged (see Textbox 2 “key findings of the interviews”): Most pregnant women, regardless of age and health status, criticized the poor quality of existing Web-based information sources and mobile apps. They had an obvious need for scientifically validated information about pregnancy-related topics. Regarding the expectations for patient engagement applications, our findings revealed a strong request for individualized feedback algorithms and individually tailored information. Several women favored more interactive apps and recommended communication platforms for both pregnant women and medical professionals. In general, usability requirements were high and the women stressed the need for a user-friendly interface in Web-based applications and mobile apps. Since many participants experienced a lack of time in the doctor-patient interaction, some suggested using Web-based applications to be better prepared for the consultation. The majority of the participants also approved of integrating digital media and modern technological devices in clinical routine and pregnancy care due to their easy-use and ubiquitous availability.

Nevertheless, several concerns emerged: a considerable number of respondents had reservations, especially concerning the safety and storage of personal data in electronic databases or applications.

Overall, no significant difference could be detected regarding user behavior and requirements in the 2 sample groups—the inpatient or outpatient participants. This could indicate that the use of eHealth or mHealth doesn’t primarily depend on the user’s health status, but instead on the socioeconomic background or education. Further research with a wider socioeconomic range in the study population is required to identify different needs.

**Key findings of the interviews.**

Key findings

- Mobile and Web-based pregnancy applications as a highly frequented source for evidence-based information about most relevant pregnancy topics
- Strong request for a more personalized output and preference for interactive applications (eg, individualized feedback algorithms, community-based features)
- Impact on doctor-patient relationship: fostering patient empowerment and a partner-like relationship
- Openness for integrating eHealth or mHealth applications in daily pregnancy care and potential digital networking among health care providers
- Data security and personal data storage in pregnancy applications as general cause for concern

### Comparison With Prior Work

Our findings are in line with other recent trials. Several studies showed that pregnant women use the Web and digital media to improve their knowledge [16,20,26,36,39] and how they evaluate evidence-based information in pregnancy applications [17]. However, previous research has reported diverging findings on the reliability of Web resources and confidence in information offered by medical professionals. Whereas Bert et al reported that 70% percent of the study participants referred to the Web as a highly reliable information source [17], our sample had a more critical stance on this. Inter alia, Kraschnewski et al found that many future mothers asked “Dr Google” first when experiencing unknown pregnancy symptoms [36]. Most of our participants first “googled” pregnancy-related symptoms, but did not experience positive reinforcement; hence, they would not recommend it.

Most of the women interviewed demanded rapid and easy access to evidence-based content on digital media. Lupton et al showed that information offered by professionals was highly valued when women had a specific, health-related concern [34]. In our sample, the need for such information was high in both inpatient and outpatient settings. Since all participants chose to deliver in a level-one prenatal center, the general concern regarding their pregnancy and their child’s health was higher.

Among others, studies from Australia and Norway reported that many pregnant women enjoyed the emotional support of other mothers through social media [19,34]. However, most of our study participants expressed reservations about online communities as a source of erroneous information. In accordance with Fredriksen et al, this may be due to the fact that the level of education in our sample was higher than average.

Relating to data security, our findings suggest that hospitalized women in particular were aware of the potential harms of applications with low data protection standards. Then again, various participants raised little concern about personal data security, which is consistent with other findings [17,22,40].

In line with other studies [22,24,34], the women in our sample appreciated interactive modules in applications. The emphasis on a personalized output of the apps could be part of the demand for a more personal support, feeling somehow they have medical support through patient-tailored applications. Lee et al showed that whereas a social networking function was important for pregnant women, interaction with health professionals still remained limited [32,36]. Standard integration of communication platforms open for lay persons and experts in such applications would provide the users with professional and instantaneous advice, leading to a more partner-like relationship.

Whereas Tripp et al assumed that reliance on health care professionals might be reduced by interactive and personalized information delivered via mobile phones [24], Ledford et al showed that no difference was detected on interpersonal clinical communication [10]. Yet, our observations showed a different result, as most women in our sample stressed the importance of individual patient-centered care. eHealth applications were seen as helpful, but more as complementary tools.

### Strengths and Limitations

As far as we know, this is the first qualitative study to analyze perceptions and expectations of pregnancy applications from the patients’ perspective in Germany, thus creating a basis for further research. This study contained a small sample of n=30, which may be considered as a limitation. However, this sample size is generally accepted for qualitative studies. Apart from ensuring richness of data through a qualitative design, the study demonstrated theoretical connectedness by using direct quotes from the original data to support themes. Although one should take care not to generalize these qualitative findings, most results were consistent with various international qualitative [19,30,36,41] and quantitative studies [14,31,34] in this field.

Nevertheless, some limitations should be taken into account. All patients were recruited at a university hospital; therefore, more patients than average had a history of two or more miscarriages (4/30, 13%). Furthermore, the average educational level was higher, potentially resulting in greater health. This needs to be considered while comparing our results to average community hospital populations. Further research among socioeconomically disadvantaged women with a lower educational level is required to identify different needs.

### Conclusions

Whereas previous research has explored the content and use of pregnancy-related applications, our study provides insight into the patients’ perceptions and expectations. We showed that pregnant women considered evidence-based information and interactive tools as the most important features. Therefore, developing medically accurate eHealth and mHealth applications poses a challenge to interdisciplinary app developers.

The next evolutionary step is to successfully integrate these evidence-based medical applications into daily health care practice, fostering both patient engagement and empowerment. Health care professionals should be committed to guiding pregnant women through these applications, exploring the ability to prevent misguiding through nonvalidated educational information, and thus reduce adverse pregnancy outcomes.

## Abbreviations

eDC: electronic Data Capture
PRO: Patient Reported Outcome
PRELAX: pregnancy application, term consists of “pregnancy” and “relax”
